# Diagnosis of common pulmonary diseases in children by X-ray images and deep learning

**DOI:** 10.1038/s41598-020-73831-5

**Published:** 2020-10-15

**Authors:** Kai-Chi Chen, Hong-Ren Yu, Wei-Shiang Chen, Wei-Che Lin, Yi-Chen Lee, Hung-Hsun Chen, Jyun-Hong Jiang, Ting-Yi Su, Chang-Ku Tsai, Ti-An Tsai, Chih-Min Tsai, Henry Horng-Shing Lu

**Affiliations:** 1 Institute of Statistics, National Chiao Tung University, Hsinchu, Taiwan; 2 Department of Pediatrics, Chang Gung Memorial Hospital, Kaohsiung Medical Centre, Kaohsiung, Taiwan; 3 Graduate Institute of Clinical Medical Sciences, College of Medicine, Chang Gung University, Taoyuan, Taiwan; 4 Department of Radiology, Chang Gung Memorial Hospital, Kaohsiung Medical Centre, Kaohsiung, Taiwan; 5 Center of Teaching and Learning Development, National Chiao Tung University, Hsinchu, Taiwan; 6 Department of Pediatric Surgery, Chang Gung Memorial Hospital, Kaohsiung Medical Centre, Kaohsiung, Taiwan

**Keywords:** Respiratory tract diseases, Mathematics and computing

## Abstract

Acute lower respiratory infection is the leading cause of child death in developing countries. Current strategies to reduce this problem include early detection and appropriate treatment. Better diagnostic and therapeutic strategies are still needed in poor countries. Artificial-intelligence chest X-ray scheme has the potential to become a screening tool for lower respiratory infection in child. Artificial-intelligence chest X-ray schemes for children are rare and limited to a single lung disease. We need a powerful system as a diagnostic tool for most common lung diseases in children. To address this, we present a computer-aided diagnostic scheme for the chest X-ray images of several common pulmonary diseases of children, including bronchiolitis/bronchitis, bronchopneumonia/interstitial pneumonitis, lobar pneumonia, and pneumothorax. The study consists of two main approaches: first, we trained a model based on YOLOv3 architecture for cropping the appropriate location of the lung field automatically. Second, we compared three different methods for multi-classification, included the one-versus-one scheme, the one-versus-all scheme and training a classifier model based on convolutional neural network. Our model demonstrated a good distinguishing ability for these common lung problems in children. Among the three methods, the one-versus-one scheme has the best performance. We could detect whether a chest X-ray image is abnormal with 92.47% accuracy and bronchiolitis/bronchitis, bronchopneumonia, lobar pneumonia, pneumothorax, or normal with 71.94%, 72.19%, 85.42%, 85.71%, and 80.00% accuracy, respectively. In conclusion, we provide a computer-aided diagnostic scheme by deep learning for common pulmonary diseases in children. This scheme is mostly useful as a screening for normal versus most of lower respiratory problems in children. It can also help review the chest X-ray images interpreted by clinicians and may remind possible negligence. This system can be a good diagnostic assistance under limited medical resources.

## Introduction

The high rates of hospitalization for acute lower respiratory infection (ALRI) among children have been highlighted^1,2^ The hospitalization rate for children with acute lower respiratory infection is 5772 per 100,000^1^. ALRI is also the leading cause of child death worldwide, accounting for 20% of mortality in children less than 5 years old^3,4^. The importance of acute lower respiratory diseases is reflected not only in the morbidity and mortality rates, but also in the long-term consequences. In developed countries, the etiology and clinical features of ALRI have been extensively investigated; however, ALRI remains a serious cause of childhood death in developing countries with an estimated 4 million deaths annually^5^. Current strategies for reducing pneumonia deaths include early detection and appropriate treatment of pneumonia. However, better diagnostic and therapeutic strategies are still urgently needed for children in low-income countries.

Bronchiolitis/bronchitis and pneumonia are the most common and significant causes of ALRI in children^6^. They are also expected to be among the four leading causes of death by 2030^7^. Bronchiolitis, a viral small airway infection, which is characterized by wide-spread inflammation of the small airways and increases in mucous production and bronchiolar epithelial cell necrosis^8^. Bronchiolitis is a clinical diagnosis characterized by tachypnea, wheezing, or crepitation in young children of less than 2 years old^9^. Bronchiolitis is a clinical diagnosis based primarily on the typical history and a physical examination of the patient. Chest radiographs may be considered in children specifically when bronchiolitis is recurrent or pneumonia is suspected. Chest radiographs for bronchiolitis can be variable and non-specific, including lung hyperinflation, peri-bronchial thickening, increased interstitial markings, and a diffuse infiltration but without a confluence consolidation or collapse^10^. As with acute bronchiolitis, acute bronchitis is a lower respiratory tract infection involving the large airways (bronchi) without evidence of pneumonia in older children. For acute bronchitis, chest X-rays are also often unclear. Thickening of the bronchial wall has been shown in some reports^11^. Although a chest radiograph is not always advised for acute bronchitis in clinical practice, a chest X-ray can help distinguish between bronchitis and pneumonia. Pneumonia is defined as a condition typically associated with fever, respiratory symptoms, and evidence of lung parenchymal involvement, either by physical examination or the presence of infiltrates in the chest radiograph. According to clinical guidelines, the gold standard for the diagnosis of pneumonia is the presence of lung infiltrates as indicated by a chest radiography^12^. Radiographically, lobar pneumonia, manifests as a non-segmental, homogeneous consolidation involving a single lobe, or less commonly, multiple lobes. Larger bronchi often remain patent with air, establishing the characteristic air bronchogram.

With recent medical developments, better diagnostic and therapeutic strategies are still urgently required for children in low-income countries. Chest radiography is the most common and important diagnostic imaging technique for pulmonary disease in clinical settings. An automated analysis can help control the variability among radiologists and advise clinicians about abnormal cases for further interpretation. Deep learning skills have been applied to the construction of models for diagnosis, such as an automated classification of pulmonary tuberculosis^13^, breast cancer detection^14^, and retinal disease detection^15^. However, most artificial-intelligence based chest X-ray schemes have focused on a single disease such as pneumonia or pneumothorax^16–18^, and there has been limited radiologist-level detection for multiple diseases based on CheXNet for adults^19^. We need a powerful system as a diagnostic tool for most of lung diseases in children. We had developed methods based on machine learning for medical image analysis^20^ and deep learning for the other types of medical images^21,22^. In this study, we used the recent development of deep learning techniques for the task of medical investigation.

The common lung diseases and chest X-ray features differ between children and adults. In clinical practice, several conditions, and not just pneumonia or pneumothorax, may be encountered by physicians. Thus, we designed a solution for a computer-aided diagnostic (CAD) scheme for chest X-ray images of several common pulmonary diseases in children.

## Materials and methods

### Design

**Figure 1:**
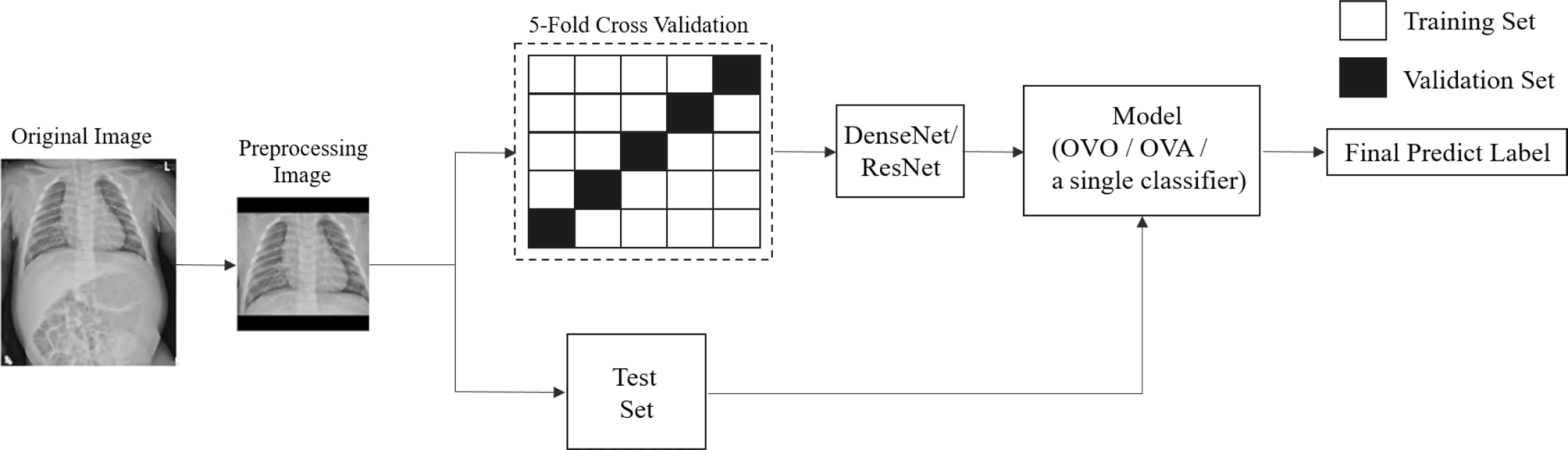
Framework of the present study. The chest X-ray images were cropped using YOLOv3 to reduce potential noise and then split into training and test set. The training set were split to conduct a fivefold cross-validation for the parameter selection. The DenseNet or ResNet algorithm was adopted to build the CNN classifier for the three different schemes. The performances of the schemes were evaluated using the test set.

This study used chest X-ray images from Kaohsiung Chang Gung Memorial Hospital and a convolutional neural network (CNN), a deep learning technique used to construct a CAD scheme. The dataset contained chest X-ray images of four different lung diseases and normal images. To deal with this problem, we used three schemes to construct the model, including a one-versus-one (OVO) scheme, one-versus-all (OVA) scheme, and a classifier trained model based on a CNN^23^. We built ten and five binary classifiers for the OVO and OVA schemes, respectively. A transfer learning model based on a residual network (ResNet)^24^ or DenseNet^25^ architecture was used to establish each binary classifier by applying our dataset, which was cropped using YOLOv3^26^. The outputs of the binary classifiers were then aggregated to predict the final output label. Finally, a test set was used to evaluate the effectiveness of the three schemes. The framework of this study is shown in Fig. 1.

### Image source

This study was conducted at the Department of Pediatrics, Kaohsiung Chang Gung Memorial Hospital, Kaohsiung, Taiwan, from January 1, 2018 to December 31, 2019 in accordance with relevant guidelines and regulations. The study was approved by the Institutional Review Board of Kaohsiung Chang Gung Memorial Hospital (201801029B0C601 and 201901277B0). Informed consent was waived by the ethics committee of Kaohsiung Chang Gung Memorial Hospital because data are decoded. This study retrospectively reviewed radiographs in children and adolescents 1–17 years in age that admitted the Department of Pediatrics, Kaohsiung Chang Gung Memorial Hospital for acute lower airway infections, pneumothorax, or other non-respiratory disease with a normal chest X-ray were recruited. All the chest X-ray images were taken for clinical demand. Each radiological interpretations was provided both by a pediatric pulmonologist and a pediatric radiologist. Radiographic images were then classified into five categories: normal, bronchiolitis/bronchitis, bronchopneumonia/interstitial pneumonitis, lobar pneumonia, or pneumothorax. All chest radiographs were taken digitally, either with a flat panel detector or with a digital storage system. Uniform and regular quality assessments were conducted on the system performance, including the display characteristics.

### Preprocessing of images

**Figure 2:**
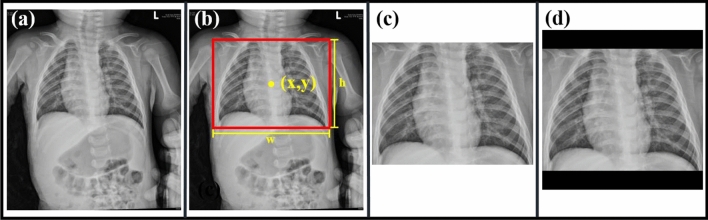
Workflow of the image preprocessing: **(a)** original image, **(b)** the location of the bounding box and a schematic of the parameters, **(c)** image cropped by the bounding box, and **(d)** image filled with black edges.

To accurately localize the relevant region of interest, we used YOLOv3 to automatically crop the original images. YOLOv3 is widely used in object detection in chest cavities. As the input of the model, an entire image was applied along with a bounding box, which is a rectangle marking the position of the desired object. There were four parameters of the bounding box: x (x coordinate of the center of the rectangle), y (y coordinate of the center of the rectangle), w (width of the rectangle), h (length of the rectangle). The trained model could predict the bounding box parameters of the desired object of the test image, which we used to frame the position of the chest cavity. For training, if the images cropped by YOLOv3 had not been square, they would have been filled with black edges. The workflow of the image preprocessing is shown in Fig. 2.

### One-versus-one (OVO) scheme

In the one versus one scheme, there were *k*(*k *− 1)/2 binary classifiers for a k-class multi-classification problem. Each binary classifier was responsible for distinguishing a different pair of categories, using only two categories of the dataset for learning. For validation, the test set was placed into all models and the corresponding outputs were aggregated to obtain the final output of the system. A weighted voting strategy was used to aggregate the output in this scheme^27^. Each binary classifier provided a predicted confidence level for the two categories. The category with the largest summed confidence was the final output.

### One-versus-all (OVA) scheme

In the one-versus-all scheme, there were *k* binary classifiers for a k-class multi-classification problem. Each binary classifier was responsible for distinguishing a specified category among all other categories. For validation, the test set was placed into all models and the corresponding outputs were aggregated to obtain the final output of the system. The maximum confidence strategy was used to aggregate the output in this scheme. Each binary classifier provided a predicted confidence for the category it focused on. The category with the largest confidence was the final output category.

### Five-fold image classification based on CNN

This study was built on fast.ai version 1.0.60 and PyTorch version 1.2.0, and using a PC with an NVIDIA GeForce GTX 1080 Ti GPU. Each classifier was constructed based on transfer learning, extracting features based on the ResNet architecture or DenseNet architecture, which replaced the fully connected layer with some randomly initialized layers such as a batch normalization layer, a dropout layer, and an activation layer. A dropout layer deactivated a certain proportion of neurons per layer to prevent an overfitting. A rectified linear unit function, which is a type of activation layer, is used for replacing a negative input with a zero to increase the nonlinearity of the model. A batch normalization layer standardized the input layer by re-centering and re-scaling to more efficiently improve the training of the neural network. There was degradation problem in some deep CNN. When the CNN was deeper, we often thought the result might be better. The degradation problem was that when CNN was deeper, the accuracy was not better or even worse. ResNet with residual learning was proposed to solve the problem. The convolutional layer connected the former 2 or 3 layer by element-wise addition to form a shortcut connection which could learn more efficiently and for solving this problem. Every convolutional layer of DenseNet was concatenated with all previous layers by channel-wise addition to form a dense connection which could reuse the low-level features. Since each layer received feature maps from all previous layers, the network could be thinner and more compact. It could compute more efficiently than ResNet. We used the two relatively new CNN models for training in this study. We used the ResNet34 architecture and DenseNet169 architecture established in fast.ai, along with our own developed dataset and focal loss^28^ for the training, where $\alpha$ and γ are the two focal loss parameters applied. The former was helpful for the problem of an imbalanced number of categories, and the latter can down-weight easy examples and thus focus the training on difficult examples. Each category was split into a training set and a test set at a ratio of 8:2 at random based on stratified sampling. The number of split images was shown in Table 1. Because our dataset was small, the training set was used to conduct a fivefold cross-validation^29^ to select hyperparameters to avoid an overfitting. The training set would be split into five sets. Regarded a set as the validation set and the others as the training set then repeated the step five times. Different sets would be regarded as the validation set each time. Used the hyperparameters with the best average performance on validation set. Finally, trained a model with the selected hyperparmeters and the original training set then used the test set to get the test performance. The hyperparameters we used for each classifier are shown in the Supplementary Table S1–S3 online.

**Table 1:** Split between training and test sets and total number for each category.

	Training set	Test set	Total number
Bronchopneumonia	676	169	845
Bronchiolitis	560	139	699
Lobar pneumonia	387	96	483
Normal	342	85	427
Pneumothorax	172	42	214
All category	2137	531	2668

### Visual explanations via gradient-weight class activation mapping (Grad-CAM)

In an image classification model, a good visual explanation means that the model can find the location of the predicted category in the test image and capture fine-grained details. Grad-CAM^30^ uses the gradient information of the last convolutional layer of the model to infer the importance of each neuron for the final decision, and the corresponding result is presented in the form of a heatmap. This tool is helpful for establishing appropriate trust in predictions from deep networks.

### Statistical analysis

In our binary classifiers, the performance was evaluated based on the total accuracy and the accuracy of each category. The accuracy was defined as the ratio of the number of correctly classified images to the total number of test images. The accuracy of each category was the accuracy of that specified category. In the binary classifiers of disease versus normal conditions, the accuracy of the disease was the same as sensitivity, and the accuracy of the normal conditions was the same as specificity. In our multiple classifiers, the performance was evaluated based on the classification rate and Cohen’s kappa^31,32^. The former was defined as the ratio of the number of correctly classified images of all categories to the number of total test images, the latter scores the successful hits independently for each class and then aggregates them, and thus is less sensitive to the randomness caused by the unbalanced amount of each category.

For all performances, we used R (version 3.6.1) and the boot package to calculate the confidence interval of the metrics, applying the BCa bootstrap method^33^ because we were unsure whether the measurements were normally distributed.

## Results

### Comparison of using or not using YOLOv3 to crop images

We constructed binary classifiers for our four disease versus normal images. Table 1 shows the number of images used for the models trained by the originals and the number of cropped images. The performance when using test images from the originals to train the disease models reached 93.99% for lobar pneumonia, 86.38% for bronchopneumonia/interstitial pneumonitis, 85.84% for bronchiolitis/bronchitis, and 92.25% for pneumothorax versus the normal images. The performance when using images trimmed by YOLOv3 was 96.69% for lobar pneumonia, 90.55% for bronchopneumonia/interstitial pneumonitis, 87.50% for bronchiolitis/bronchitis, and 94.49% for pneumothorax. The details of the performance are shown in Table 2.

**Table 2:** Diagnostic performance of binary classifiers for diseases versus normal conditions built using the original images and cropped images, where the numbers of test images are as listed in Table 1.

Type	Binary classifier	Performance		
	Category	Accuracy	Sensitivity	Specificity
Original images	Bronchiolitis	85.84%	89.21%	80.46%
		(0.8029–0.8982)	(0.8273–0.9394)	(0.6980–0.8750)
	Bronchopneumonia	86.38%	88.82%	81.61%
		(0.8054–0.8988)	(0.8323–0.9321)	(0.7210–.8915)
	Lobar pneumonia	93.99%	94.79%	93.10%
		(0.8962–0.9627)	(0.8823–0.9796)	(0.8509–0.9749)
	Pneumothorax	92.25%	80.95%	97.26%
		(0.8529–0.9535)	(0.6600–0.9111)	(0.9212–1.0000)
Cropped images	Bronchiolitis	87.50%	89.21%	84.71%
		(0.8259–0.9063)	(0.8280–0.9328)	(0.7590–0.9162)
	Bronchopneumonia	90.55%	91.72%	88.24%
		(0.8622–0.9331)	(0.8710–0.9545)	(0.8049–0.9412)
	Lobar pneumonia	96.69%	96.88%	96.47%
		(0.9194–0.9834)	(0.9145–0.9900)	(0.9065–0.9884)
	Pneumothorax	94.49%	90.48%	96.47%
		(0.8818–0.9685)	(0.9026–0.9892)	(0.8922–0.9886)

**Figure 3:**
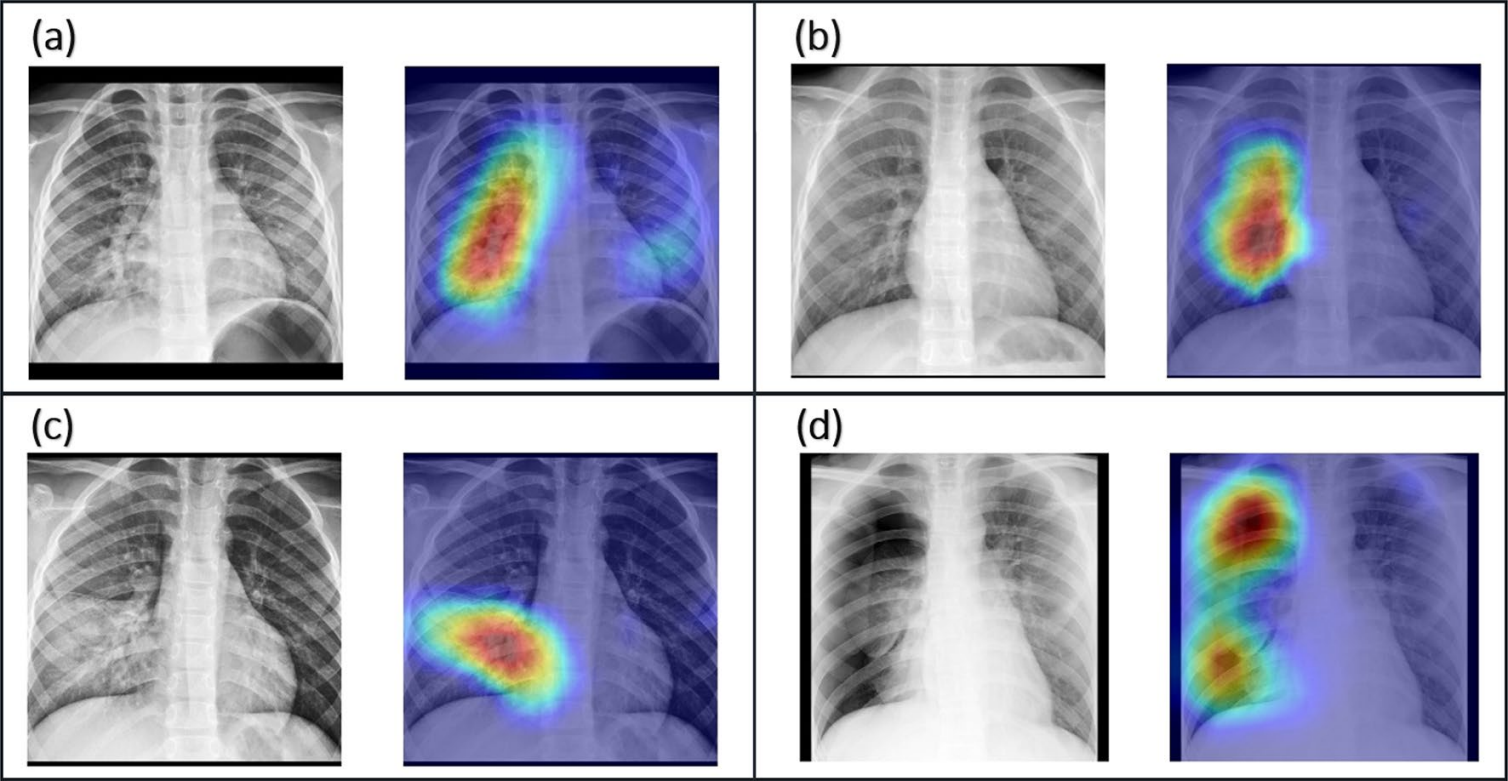
Image pairs of radiographs and the corresponding Grad-CAM of the test set: **(a)** bronchopneumonia, **(b)** bronchiolitis, **(c)** lobar pneumonia, **(d)** pneumothorax.

The performance of the models trained using the cropped images was better than that with the original images. As Table 2 showed, the accuracy was generally higher and the corresponding confidence intervals were narrower. The cropped images for training had resulted in higher sensitivity or specificity. We hoped that the clinical features could be focused in Grad-CAM. The regions captured by the model trained with the cropped images were shown in Fig. 3. Based on the above experiment, we used the trimmed images for the subsequent study.

### Performance of multiple classification

In clinical, unexpected condition may be encountered by physicians, we would like to expand the binary classifiers to multi-class classifier since only using binary classifiers needed some prior assumptions. For example, a new X-ray image would be put in the binary classifier of pneumothorax versus normal if it was assumed to be pneumothorax. To find single solution for five categories of the common pediatric lower airway problems, we investigated three schemes combined with a deep learning technique. The number of images used is shown in the last row of Table 1. A total of 531 test set images were used, which contained 169 bronchopneumonia images, 139 bronchiolitis images, 96 lobar pneumonia images, 42 pneumothorax images, and 85 normal images.

**Figure 4:**
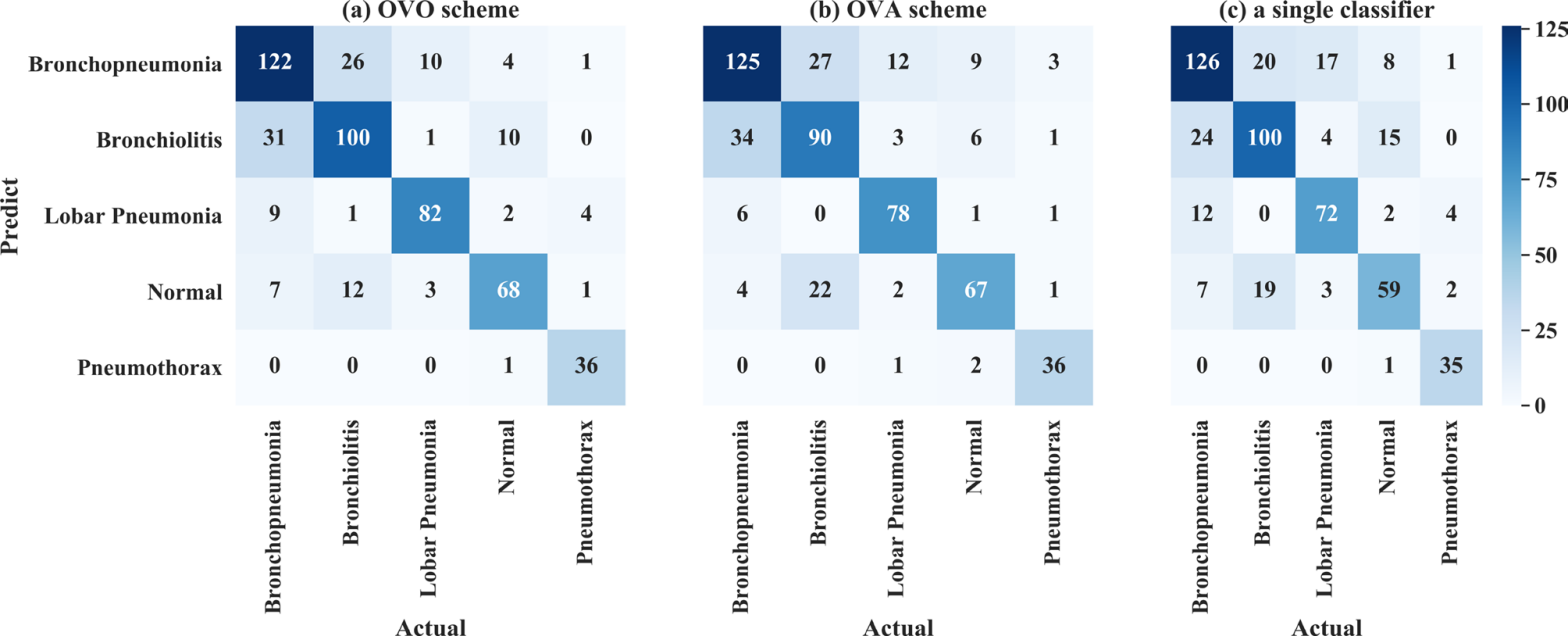
Confusion matrix of **(a)** OVO scheme, **(b)** OVA scheme, and **(c)** simple classifier.

First, the OVO scheme used the output of the ten binary classifiers to aggregate the final output. The performance of the ten binary classifiers was shown in Supplementary Table S4 online and reached almost over 90%. No gaps were shown between the accuracies of the two categories for the binary classifiers, which indicates that the classifiers did not tend to learn the features of a specified category. The aggregated results based on the OVO scheme achieved a classification rate of 76.84% and a Cohen’s Kappa score of 69.76%. From the confusion matrix in Fig. 4a, bronchopneumonia and bronchitis were easily misclassified.

Second, the accuracy of the binary classifiers of the OVA scheme were all over 80%, as shown in the Supplementary Table S5 online, although the accuracy of the “other” category was consistently higher than that of each specified category. The aggregated results of the OVA scheme reached a classification rate of 74.58% and a Cohen’s Kappa score of 66.74%. From the confusion matrix shown in Fig. 4b, bronchopneumonia and bronchiolitis were easily misclassified, and bronchiolitis was easy misclassified as normal.

Finally, the simple classifier achieved a classification rate of 73.82% and a Cohen’s Kappa of 65.70%. From the confusion matrix shown in Fig. 4c, except for pneumothorax, which might be easily distinguished from the other conditions, the proportion of correct classifications for the other diseases was not high. In detail, there were three pairs that were not easy to identify: bronchiolitis and bronchopneumonia, bronchiolitis and normal, and bronchopneumonia and lobar pneumonia.

**Figure 5:**
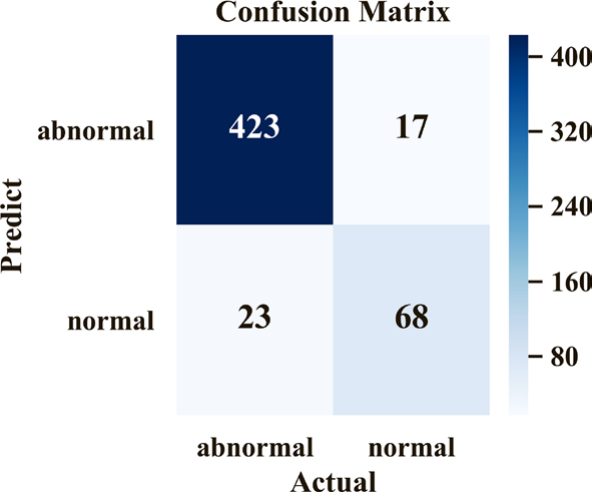
Confusion matrix of diagnosis of lung diseases or normal conditions.

As Table 3 shows, the OVO scheme achieved the best results among the three approaches, with the highest classification rate of 76.84% and a Cohen’s Kappa of 69.76%. Under this scheme, the proposed model could diagnose whether a patient has a lung disease with 92.47% accuracy, 90.77% sensitivity, and 80.00% specificity; the corresponding confusion matrix is shown in Fig. 5.

**Table 3:** Performances of OVO and OVA schemes and a simple classifier.

	OVO	OVA	Simple classifier
Bronchopneumonia	72.19%	73.96%	74.56%
Bronchiolitis	71.94%	64.75%	71.94%
Lobar pneumonia	85.42%	81.25%	75.00%
Normal	80.00%	78.82%	69.41%
Pneumothorax	85.71%	85.71%	83.33%
Classification rate	76.84% (0.7274–0.8001)	74.58% (0.7081–0.7815)	73.82% (0.7024–0.7759)
Cohen’s kappa	69.76% (0.6465–0.7458)	66.74% (0.6143–0.7197)	65.70% (0.6103–0.7051)

## Discussion

In resource-rich countries, the annual incidence of pneumonia in children is estimated to be 1.5 to 3 per 1000^34^. Approximately one-half of children younger than 5 years of age with community-acquired pneumonia require hospitalization^2^. In a systematic review, the annual incidence of pneumonia in children younger than 5 years in age from resource-limited countries in 2015 was estimated to be 231 per 1000, with 50–80% of children having severe pneumonia requiring hospitalization^2^. Chest radiographs are required for confirmation/exclusion of the diagnosis in children with clinical evidence of pneumonia.

Community acquired pneumonia can be divided into three distinctive patterns through imaging examinations, namely, consolidation (lobar pneumonia), peribronchial nodules (bronchopneumonia), and ground-glass opacity (interstitial pneumonia). In this study, we divided our pneumonia images into two groups (bronchopneumonia/interstitial pneumonitis and lobar pneumonia) because we found that peribronchial infiltration and ground-glass opacity often coexist in the case of pneumonia. This phenomenon has also been described in a previous report^36^.

Bronchopneumonia is radiographically identified by its patchy appearance with peribronchial thickening and ill-defined air-space opacities. As the illness becomes more severe, consolidation involving the terminal and respiratory bronchioles and alveoli results in the development of centrilobular nodular opacities or air-space nodules. The consolidation can develop further and coalesce to give a lobular or lobar pattern of involvement. Unlike lobar pneumonia, which starts in the alveoli, bronchopneumonia starts in the airways as acute bronchitis. This can explain the relative inaccuracy in differentiating between bronchitis and bronchopneumonia.

Although chest radiography is considered the best method for diagnosing pneumonia, a radiographic evaluation is subjective and inconsistencies are found in the interpretation among different radiologists of the same chest radiograph^35,36^. Significant inconsistencies exist for minor changes and in the description of the infiltrates, although the agreement regarding the presence or absence of a consolidation/ infiltrates was high. Levels of disagreement were highest for children of less than 5 years in age^34^. For the radiographic findings, a significant inter-observer variability was determined in the interpretation of patchy (48.8%) and perihilar (28.1%) changes.

In general, it is difficult to determine a specific pathogen for pneumonia based solely on imaging findings. However, a radiographic image can help confirm the diagnosis of pneumonia^36^. Imaging studies also play an auxiliary role in evaluating the effectiveness of medical treatment. We provided a method for cropping a cavity automatically. Another study reviewed previous methods for localizing a lung region^37^. Because the features might not only occur in the lung, we tried to focus on the cavity and then applied YOLOv3 to train our own model for use. With this model, we can save a significant amount of time because it is no longer necessary to crop images by hand.

The methods focusing on a single disease often achieved a good performance. Approaches developed by Liang and Zheng^16^ and Saraiva et al.^17^ obtained accuracy over 90% and Taylor et al.^18^ achieved an accuracy of over 90% AUC on their own dataset and over 80% AUC on an external dataset. As shown in Table 2, we also obtained more than 90% of accuracy, sensitivity, and specificity for both pneumonia and pneumothorax. Nearly 90% accuracy was also obtained for the other two diseases. However, from a clinical perspective, several conditions, and not just pneumonia or pneumothorax, may be encountered by physicians. Based on this dilemma, we designed a holistic method for diagnosing the common diseases for children, and we attempted three different schemes for such an aim. Among these three schemes, the OVO scheme achieved the best results. A study comparing the OVO scheme and the OVO scheme under different classifier methods was also conducted^23^ but did not include classifiers based on deep learning. The results showed that the performance of the OVO scheme is typically better than that of the OVA scheme, and we obtained a similar conclusion. In our study, binary classifiers of the OVO scheme mostly performed well, and thus the aggregation also likely achieved good results. The unbalanced number of different categories might have caused the OVA scheme to perform poorly. In the simple classifier, the unbalanced data were also important. The radiographic findings were different in terms of degree in certain diseases and some patients might have had subtle radiographic findings, thereby causing the simple classifier to perform poorly because it had to learn the features of each category simultaneously.

From the confusion matrix of the three schemes, we found that bronchopneumonia and bronchiolitis are easier misclassified than other disease. This is not surprising because there is substantial inter-observer variability in the reporting of chest radiographs particularly in young children with pneumonia by radiologists^36^. The radiographic findings in acute bronchiolitis/bronchitis include hyperinflation, patchy areas of consolidation or atelectasis, streaky perihilar opacities or tram tracks due to bronchial wall or interstitial thickening, and reticular or reticulonodular opacities. Bronchopneumonia begins with airway mucosa infection and subsequently extends into the adjacent alveoli, the bronchopneumonia pattern consists of multiple areas of patchy consolidation, often bilaterally, lack of air bronchograms and progressive coalescence of the patchy consolidation with time. During the early disease stage of bronchopneumonia, there are similar findings of acute bronchiolitis/bronchitis and bronchopneumonia^38^. A previous study conducted for chest radiography of pediatric pneumonia observed wide variability in the interpretation of chest radiographs among radiologists. The inter‐rater reliability for alveolar infiltration demonstrated substantial reliability (κ = 0.69) and less reliability (κ = 0.14) for interstitial infiltration. Similarly, the intra‐rater assay for interstitial infiltration also demonstrated a wide variability and less reliable result^39^. Strengthening the classification of these two diseases is an important area of future study. Clinical data may help in diagnosing between the two diseases, and we may consider applying a two-stage classification.

Here we have constructed a computer-aided scheme by deep learning for common pulmonary diseases in children. This scheme is mostly useful as a screening for normal versus most of lower respiratory problems in children. It can also help review the chest X-ray images interpreted by clinicians and may remind possible negligence. This system can be a good diagnostic assistance under limited medical resources.

## Supplementary information

Supplementary Information.
