# Supplementary material for: Diagnosis of common pulmonary diseases in children by X-ray images and deep learning
Source: Sci Rep. 2020 Oct 15;10:17374. doi: 10.1038/s41598-020-73831-5 (PMC7566516; doi:10.1038/s41598-020-73831-5)
Supplement: Supplementary file 1 — Supplementary Information. [file 41598_2020_73831_MOESM1_ESM.pdf]

Supplementary information

Diagnosis of common pulmonary diseases in children by X-ray images and deep learning

Kai-Chi Chen[1+], Hong-Ren Yu[2,3+], Wei-Shiang Chen[1], Wei-Che Lin[4], Yi-Chen Lee[2,3], Hung-Hsun Chen[5], Jyun-Hong Jiang[6], Ting-Yi Su[1], Chang-Ku Tsai[2,3], Ti-An Tsai[2,3], Chih-Min Tsai[2,3], Henry Horng-Shing Lu[1*]

[1] Institute of Statistics, National Chiao Tung University, Taiwan
[2] Department of Pediatrics, Chang Gung Memorial Hospital - Kaohsiung Medical Centre, Kaohsiung, Taiwan
[3] Graduate Institute of Clinical Medical Sciences, College of Medicine, Chang Gung University, Taiwan
[4] Department of Radiology, Chang Gung Memorial Hospital - Kaohsiung Medical Centre, Kaohsiung, Taiwan
[5] Center of Teaching and Learning Development, National Chiao Tung University, Taiwan
[6] Department of Pediatric Surgery, Chang Gung Memorial Hospital - Kaohsiung Medical Centre, Kaohsiung, Taiwan
* corresponding author hslu@stat.nctu.edu.tw
[+] these authors contributed equally to this work

Supplementary Table S1: Hyperarameters used in the binary classifiers of OVO scheme

Binary Classifier		Network and Parameters				
Category1	Category2	Network	Batch	Epoch	α	γ
Bronchiolitis	Normal	ResNet34	16	7	1	0
Bronchopneumonia	Normal	ResNet34	16	9	2	0
Lobar Pneumonia	Normal	ResNet34	16	7	1	0
Pneumothorax	Normal	ResNet34	16	7	0.5	1
Bronchiolitis	Pneumothorax	ResNet34	16	5	3	2
Bronchopneumonia	Pneumothorax	ResNet34	16	7	4	0
Pneumothorax	Lobar Pneumonia	ResNet34	16	7	0.5	0
Bronchiolitis	Lobar Pneumonia	ResNet34	16	7	1.5	1
Bronchopneumonia	Lobar Pneumonia	ResNet34	16	11	4.5	0
Bronchiolitis	Bronchopneumonia	DenseNet169	16	13	1	0

Supplementary Table S2: Hyperparameters used in the binary classifiers of OVA scheme

Binary Classifier		Network and Parameters				
Category1	Category2	Network	Batch	Epoch	α	γ
Bronchopneumonia	Other	DenseNet169	32	9	0.5	1
Bronchiolitis	Other	DenseNet169	32	11	0.5	2
Lobar Pneumonia	Other	DenseNet169	32	13	0.25	2
Normal	Other	DenseNet169	32	11	0.2	0
Pneumothorax	Other	DenseNet169	32	7	0.1	1

Supplementary Table S3: Hyperparameters used in the multiple classifier

	Network and Parameters		
	Network	Batch	Epoch
Multiple Classifier	DenseNet169	32	13

Supplementary Table S4. The details of the performance of the binary classifiers in OVO scheme.

Binary Classifier		Performance		
Category1	Category2	Accuracy	Accuracy of Category1	Accuracy of Category2
Bronchiolitis	Normal	87.50% (0.8259 - 0.9063)	89.21% (0.8280 - 0.9328)	84.71% (0.7590 - 0.9162)
Bronchopneumonia	Normal	90.55% (0.8622 - 0.9331)	91.72% (0.8710 - 0.9545)	88.24% (0.8049 - 0.9412)
Lobar Pneumonia	Normal	96.69% (0.9194 - 0.9834)	96.88% (0.9145 - 0.9900)	96.47% (0.9065 - 0.9884)
Pneumothorax	Normal	94.49% (0.8818 - 0.9685)	90.48% (0.9026 - 0.9892)	96.47% (0.8922 - 0.9886)
Bronchiolitis	Pneumothorax	97.24% (0.9337 - 0.9890)	98.56% (0.9411 - 1.0000)	92.86% (0.7962 - 0.9773)
Bronchopneumonia	Pneumothorax	97.63% (0.9384 - 0.9858)	98.82% (0.9561 - 1.0000)	92.86% (0.7859 - 0.9783)
Lobar Pneumonia	Pneumothorax	95.65% (0.8986 - 0.9783)	97.92% (0.9289 - 1.0000)	90.48% (0.7712 - 0.9722)
Bronchiolitis	Lobar Pneumonia	95.74% (0.9234 - 0.9745)	96.40% (0.9233 - 0.9869)	94.79% (0.8843 - 9807)
Bronchopneumonia	Lobar Pneumonia	90.94% (0.8604 - 0.9358)	93.49% (0.8916 - 0.9646)	86.46% (0.7800 - 0.9237)
Bronchiolitis	Bronchopneumonia	80.52% (0.7565 - 0.8442)	81.66% (0.7470 - 0.8716)	79.14% (0.7022 - 0.8500)

Supplementary Table S5. The details of the performance of the binary classifiers in OVA scheme.

Binary Classifier		Performance		
Category1	Category2	Accuracy	Accuracy of Category1	Accuracy of Category2
Bronchiolitis	Other	83.05% (0.7872 - 0.8512)	66.91% (0.5518 - 0.7123)	88.76% (0.8553 - 0.9184)
Bronchopneumonia	Other	81.17% (0.7853 - 0.8512)	66.86% (0.6356 - 0.7748)	87.85% (0.8356 - 0.9081)
Lobar Pneumonia	Other	94.35% (0.9209 - 0.9591)	81.25% (0.7197 - 0.8854)	97.24% (0.9547 - 0.9844)
Normal	Other	90.02% (0.8682 - 0.9209)	74.12% (0.6378 - 0.8231)	93.05% (0.9057 – 0.9508)
Pneumothorax	Other	97.74% (0.9587 - 0.9868)	73.81% (0.5794 - 0.8516)	99.80% (0.9894 – 1.0000)
